# Socioeconomic disparities in Rwanda’s under-5 population’s growth tracking and nutrition promotion: findings from the 2019–2020 demographic and health survey

**DOI:** 10.1186/s12887-023-04284-8

**Published:** 2023-09-16

**Authors:** Michael Ekholuenetale, Osaretin Christabel Okonji, Chimezie Igwegbe Nzoputam, Clement Kevin Edet, Anthony Ike Wegbom, Amit Arora

**Affiliations:** 1 Department of Epidemiology and Medical Statistics, Faculty of Public Health, College of Medicine, University of Ibadan, Ibadan, 200284 Nigeria; 2 School of Pharmacy, University of the Western Cape, Cape Town, 7530 South Africa; 3 Department of Public Health, Center of Excellence in Reproductive Health Innovation (CERHI), College of Medical Sciences, University of Benin, Benin City, 300001 Nigeria; 4 Department of Medical Biochemistry, School of Basic Medical Sciences, University of Benin, Benin City, 300001 Nigeria; 5 Department of Community Medicine, College of Medical Sciences, Rivers State University, Port Harcourt, 500101 Nigeria; 6 Department of Public Health Sciences, College of Medical Sciences, Rivers State University, Port Harcourt, 500101 Nigeria; 7 Translational Health Research Institute, Western Sydney University, Campbelltown, NSW 2560 Australia; 8 School of Health Sciences, Western Sydney University, Penrith, NSW 2751 Australia; 9 Health Equity Laboratory, Campbelltown, NSW 2560 Australia; 10 Discipline of Child and Adolescent Health, The Children’s Hospital at Westmead Clinical School, Faculty of Medicine and Health, The University of Sydney, Westmead, NSW 2145 Australia; 11 Oral Health Services, Sydney Local Health District and Sydney Dental Hospital, NSW Health, Surry Hills, NSW 2010 Australia

**Keywords:** First 2000 days, Malnutrition, Stunting, Socioeconomic inequalities, Growth monitoring, Nutrition promotion

## Abstract

**Background:**

Regular growth monitoring can be used to evaluate young children’s nutritional and physical health. While adequate evaluation of the scope and quality of nutrition interventions is necessary to increase their effectiveness, there is little research on growth monitoring coverage measurement. The purpose of this study was to investigate socioeconomic disparities in under-5 Rwandan children who participate in growth monitoring and nutrition promotion.

**Methods:**

We used data from the 2019–2020 Rwanda Demographic and Health Survey (RDHS), which included 8092under-5 children. Percentage was employed in univariate analysis. To examine the socioeconomic inequalities, concentration indices and Lorenz curves were used in growth monitoring and nutrition promotion among under-5 children.

**Results:**

A weighted prevalence of 33.0% (95%CI: 30.6-35.6%) under-5 children growth monitoring and nutrition promotion was estimated. Growth monitoring and nutrition promotion among under-5 children had higher uptake in the most disadvantaged cohort, as the line of equality sags below the diagonal line in Lorenz curve. Overall, there was pro-poor growth monitoring and nutrition promotion among under-5 in Rwanda (Conc. Index = 0.0994; SE = 0.0111). Across the levels of child and mother’s characteristics, the results show higher coverage of under-5 growth monitoring and nutrition promotion in the most socioeconomic disadvantaged cohort.

**Conclusion:**

The study found a pro-poor disparity in growth monitoring and nutrition promotion among under-5 children in Rwanda. By implication, the most disadvantaged children had a higher uptake of growth monitoring and nutrition promotion. The Rwanda government should develop policies and programmes to achieve the universal health coverage for the well-off and underserved population.

## Background

The United Nations International Children’s Emergency Fund (UNICEF) defines growth monitoring as a monthly assessment of a child’s development in terms of growth with reference to the World Health Organization (WHO) benchmark, using anthropometric indicators to detect growth dysfunction and malnutrition threshold [1, 2]. Child growth monitoring is a useful practice to evaluate the health and nutritional status of children [3]. Several indicators such as stunting, underweight, wasting, undernutrition and overweight can be measured during child growth monitoring [4]. However, none of these indicators are exactly the same. For example, stunting is not always same as undernutrition [5]. The foundational elements for healthy growth, a strong immune system and the development of the brain are all built on a child’s first year of nutrition. It also helps prevent noncommunicable diseases (NCDs) linked to obesity in the future [6, 7]. Despite significant recent progress in reducing child mortality, over five million children die before age five every year, mainly as a result of inadequate infant and young child nutrition (IYCN) [8]. An alarming amount of food insecurity has resulted in 144 million stunted children and nearly half of all under-5 anaemia worldwide [9].

Undernutrition accounts for approximately 45% of deaths in under-5 children globally [10]. The majority of children’s suboptimal feeding occurs in resource-constrained settings. Furthermore, the prevalence of childhood malnutrition are rising in resource-constrained countries. For example, approximately 45.4 million children were estimated to be wasted, 38.9 million children were overweight and 149.2 million under-5 had stunted growth [11]. Children who are stunted are becoming less common across all WHO regions, except in African [11]. However, with regard to obesity, roughly half of all countries have either seen no improvement or a worsening of the situation [11].

Growth monitoring and nutrition promotion (GMNP) is a preventive strategy that advocates for appropriate and proper feeding practices for under-5 children and monitors, measures, interprets and analyses potential causes of adequate or insufficient child growth. Additionally, it encourages interaction and communication, promotes appropriate health-seeking behaviour, child’s nutritional status and reduces child morbidity and mortality [1, 3, 12]. Several countries around the world have very low rates of attendance and promotion toward GMNP and many caregivers have poor understanding of the growth charts. The GMNP programme implementation and subsequent changes in care practices have not been extensively studied in many countries [13, 14]. It is challenging to carry out effective growth monitoring activities and community involvement are frequently ignored when determining whether to include growth monitoring in national surveillance programmes [14].

The Sustainable Development Goals (SDGs), specifically those targeting to eradicate poverty in all of its forms globally (SDG 1), eradicate hunger, achieve food security, improve nutrition and promote sustainable agriculture (SDG 2), as well as ensuring healthy lives and promoting health and quality of life for all at all ages (SDG 3), must be attained adequately by reducing childhood malnutrition [15, 16]. Several countries have agreed to the global targets to reduce stunting (chronic undernutrition) by 40% by 2025 and to keep the prevalence of wasting (acute undernourishment) in children under the age of five to less than 5% [17]. The practices of GMNP among key population such as under-5 children are key in achieving these SDGs. Taking into account global efforts to improve infant and child feeding practices through the International Code of Marketing of Breast milk Substitutes, the promotion of proper nutrition, including breastfeeding [18], the Global Strategy for Infant and Young Child Feeding [19] and The Code, the baby friendly hospital initiative (BFHI) [20], are essential part of children’s growth mechanisms.

The UNICEF conceptual framework on nutrition, posited that psychosocial stimulation, nutrition and health are the critical components for improving and enhancing children’s quality of life. This implies that appropriate feeding practices must be accelerated to achieve better growth and development [17]. Due to extreme financial uncertainty that resulted in Rwanda’s genocide nearly three decades ago, malnutrition has been said to be more prevalent [21]. Several progressive policies outlined in Rwanda’s Vision 2020 plan have reportedly been put into practice to support the country’s economic recovery [22]. In turn, this has led to significant improvements in health of the populace across a range of population health metrics [23]. For instance, between 2000 and 2015, the rates of newborn and under-5 mortality decreased, while the rate of vaccinations significantly increased [24]. This progress might be attributed to advancements from citizens’ participation to improve the healthcare system, such as the implementation of the neighbourhood health insurance policy to enhance economic access to care and the development of a strong health-care workforce.

Research to investigate socioeconomic inequalities in GMNP among Rwanda’s under-5 population have, to the best of our knowledge, received little or no attention in spite of several studies conducted thus fa on the subject matter [23–25]. The dearth of under-5 GMNP data in Rwanda is a critical gap that our study is set out to fill. Therefore, we would like to answer the question of who, between the disadvantaged or well-off are more likely to uptake under-5 GMNP. The findings from this study add to knowledge base or literature and useful for stakeholders in healthcare system to develop viable interventions and adopt relevant policies. The magnitude of these inequalities is investigated as it helps to reduce inequalities in service uptake. The objective of this study was to evaluate socioeconomic inequalities in under-5 GMNP in Rwanda.

## Methods

### Data source

Data from children’s survey questionnaire from the 2019–20 Rwanda Demographic and Health Survey (RDHS) was analysed in this study. A total of 8,092 under-5 children were included in the sample. The 1992, 2000, 2005, 2010, and 2014–15 surveys were followed by the 2019–20 RDHS, which was the sixth round. The survey was conducted by the Rwandan National Institute of Statistics with funding from the Inner-City Fund (ICF) and the Ministry of Health. The survey was conducted from November 2019 to July 2020. Data collection was suspended for about three months (March-June) due to the effects of the lockdown that followed the coronavirus pandemic in 2020 [26]. Information relevant to monitoring population health was gathered by the RDHS on topics including nutrition among others [26]. A previous study has reported the methodology of RDHS [27].

### Sampling design

An entire nation-wide sampling frame of enumeration areas (EAs) was provided by the National Institute of Statistics, the RDHS’s implementing organization. The first step in the 2-stage stratified cluster sampling approach was to select clusters made up of EAs. There were 500 clusters, with 388 in rural and 112 in urban areas. In the second phase, systematic household sampling was carried out. A household listing was done in each of the selected EAs from June to August 2019, and the households that were surveyed were selected at random. With an average of 26 households per cluster across the nation, there were 13,000 households.

### Selection and measurement of variables

#### Outcome

Participation in growth monitoring and nutrition promotion services was estimated in this study and measured dichotomously as “1” if “yes” and “0” otherwise. This outcome variable has also been measured by a previous study [25].

#### Explanatory variables

There are several variables that were included in this study, thus: age of mother, family mobility, mother’s education, mother’s marital status, currently pregnant, currently breastfeeding, mother’s employment status, child’s age (months), sex of child, preceding birth interval, place of childbirth, geographical region. In addition, low birthweight (<2.5 kg) compared to normal birthweight (≥2.5 kg); male versus female household head; household wealth is divided into five quintiles, from poorest to richest; urban versus rural status of residence; households with 1–4, 5–6, and 7 + members; Furthermore, items such as rural residence, lowest household wealth level, mothers with no formal education and not working were used to compute the socioeconomically disadvantaged level. To separate the overall assigned scores to low, moderate, and high, the standardized z-score was subjected to principal component analysis (PCA).

#### Concentration curves and indices

The concentration index is a widely used method for analyzing health inequities. The indices and curves investigate the presence of health inequalities. They do not, however, quantify the degree of health disparities. The Erreygers normalised concentration indices [28] were used in this study to assess the degree of socioeconomic disparities in tracking growth and promoting nutrition. Among the several indices that may have been employed, the Erreygers was chosen because of its simplicity and capacity to be decomposable.

The concentration index can be computed making use of the ‘convenient covariance’ as shown below:

1 $$CI = \frac{2}{\hat y} COV\,({y_i},{R_i})$$

Where: y_i_ is the health variable.

ŷ is the mean of y_i_.

R_i_ is the fractional rank of the ith individual.

COV symbolizes the covariance.

Concentration indices are calculated by dividing the area between the concentration curve and the line of equality (the 45-degree line) by two [29]. A concentration curve on the 45° line indicates that there is no health inequity. The concentration curve’s distance from the line of equality (45° line) indicates the magnitude of the health inequality. The wider the distance between the concentration curve and the line of equality, the higher the level of health inequity. This study chose to employ the normalized formulae because it is suggested that normalizing the health concentration index formula assures that the boundaries issue for a binary Cardinal Health variable is resolved. The Erreygers normalized index (E(c)) is denoted as:

2 $${E_c} = \frac{4\hat y} {{{y^{max }} - {y^{min }}}}CI$$

In the case of binary variables, y^max^ - y^min^ represents the range of the health variable, which is ‘one’. As both corrected concentration indices are extensively used in the health literature, the current investigation concentrated on the Erreygers normalised index.

#### Decomposing the erreygers normalised concentration index

The Erreygers Normalised concentration index can be decomposed to calculate the contributions of maternal health indicator determinants [30, 31]. Health inequalities were decomposed into the contributions of several explanatory factors, with each contribution being the product of health elasticity. Given a linear relationship between individual health (yi) and a collection of k explanatory variables, yi will be as follows:

3 $${y_i} = a + \sum\nolimits_k {{\beta _k}{X_{ki}} + {\varepsilon _i}}$$

Wagstaff et al. [31] demonstrated that the concentration index for any health measure that has a linear relationship with a set of k exploratory variables may be divided as follows:

4 $$CI = \sum\limits_k {\left( {\frac{{{\beta _k}{{\dot {\rm X}}_k}}}{\hat y}} \right)} C{I_k} + \frac{{GC{I_\varepsilon }}}{\hat y}$$

Where: β_k is_ the partial.

ŷ is the mean of the health variable.

ẋ_k_ is the mean of ẋ_k_.

CI_k_ denotes the concentration index of x_k_ against income.

GC_ε_ is the generalised concentration for the error term.

To decompose the Erreygers concentration index, we modied Eq. (4) as shown below [32]:

5 $${E_c} = 4\left[ {\sum\limits_k {({\beta _k}{{\dot {\rm X}}_k})C{I_k} + GC{I_\varepsilon }} } \right]$$

### Statistical analysis

The survey module (‘svy’) command was used to adjust for sampling design. Percentage was used in the univariate analysis. To examine socioeconomic inequalities, concentration indices and curves were used in tracking growth and promoting nutrition. The concentration index value is positive when growth monitoring and nutrition promotion were higher in high socioeconomic disadvantaged children. The converse is however true when the concentration index value is negative [4, 33]. The level of statistical significance was set at p < 0.05. For data analysis, Stata version 14 (StataCorp., College Station, TX, USA) was utilized.

### Ethical consideration

For the purposes of this study, identifier information was removed from a secondary dataset that was publicly accessible. In order to get the respondent’s informed consent, RDHS adhered to a recognised ethical procedure. Since the authors were granted approval for this study dataset, no additional participants’ consent was required. You can find information about DHS ethical standards here: http://goo.gl/ny8T6X.

## Results

A weighted prevalence of 33.0% (95%CI: 30.6-35.6%) under-5 GMNP was estimated. It follows that in 2019–20, approximately two-thirds of Rwandan under-5 children did not utilize growth monitoring and nutrition promotion services.

Table 1 shows the distribution of under-5 GMNP across child and mother characteristics. Based on the results, the most disadvantaged children had higher prevalence in the uptake of under-5 GMNP in Rwanda. The prevalence of under-5 GMNP increased as children get older. Similarly, normal birthweight (≥2.5 kg) under-5, female folks, those delivered at health facility, native, those having mothers with no formal education, who listen to radio or currently in union, covered by health insurance or from households with male headship, resident in South, West region or rural areas, reported higher prevalence of under-5 GMNP respectively.

**Table 1:** Distribution of under-5 GMNP in Rwanda across socioeconomic disadvantaged level

Variable	n (%)	Socioeconomic disadvantaged level		
		Least disadvantaged (n = 2707)	Moderate disadvantaged (n = 2703)	Most disadvantaged (n = 2682)
**Child’s age (months)**				
0–11	1618 (20.0)	22.0	31.2	34.7
12–23	1633 (20.2)	25.0	40.2	43.5
24–35	1643 (20.3)	26.0	43.9	38.8
36–47	1633 (20.2)	26.4	39.2	39.4
48–59	1565 (19.3)	30.6	40.2	46.1
P		0.240	0.016*	0.018*
**Preceeding birth interval**				
< 24 months	867 (10.7)	25.2	38.9	40.0
24–48 months	3618 (44.7)	27.4	40.6	43.8
> 48 months	1527 (18.9)	27.1	37.9	38.6
First born	2080 (25.7)	19.9	30.7	32.9
P		0.028*	0.012*	0.005*
**Birthweight (kg)**				
< 2.5	541 (7.1)	28.3	34.7	31.9
≥ 2.5 (normal weight)	7118 (92.9)	24.7	38.2	41.0
P		0.441	0.499	0.087
**Sex of child**				
Male	4095 (50.6)	23.7	35.6	39.4
Female	3997 (49.4)	25.6	39.1	40.7
P		0.411	0.154	0.619
**Place of delivery**				
Health facility	7663 (94.7)	25.0	37.7	40.0
Home	429 (5.3)	5.3	28.1	40.0
P		0.048*	0.121	0.995
**Mother’s age (years)**				
15–24	1287 (15.9)	24.3	27.3	31.1
25–34	3949 (48.8)	23.9	37.4	41.5
35+	2856 (35.3)	26.5	43.0	43.5
P		0.602	< 0.001*	0.001*
**Family motility**				
< 5 years	2791 (34.5)	20.8	26.6	37.2
5 + years (native)	5301 (65.5)	28.4	42.8	41.2
P		0.001*	< 0.001*	0.147
**Mother’s education**				
No formal education	918 (11.3)	31.3	42.8	47.7
Primary	5277 (65.2)	26.8	37.3	39.6
Secondary+	1897 (23.4)	21.8	34.9	29.7
P		0.050	0.299	< 0.001*
**Household size**				
1–4	3084 (38.1)	23.2	35.9	38.1
5–6	3154 (39.0)	24.7	38.6	44.2
7+	1854 (22.9)	27.0	37.1	36.4
P		0.435	0.633	0.024*
**Mother read newspaper**				
Disagree	6528 (80.7)	25.1	35.3	39.7
Agree	1564 (19.3)	23.8	47.2	42.2
P		0.595	< 0.001*	0.492
**Mother listen to radio**				
Disagree	1935 (23.9)	23.1	33.6	35.4
Agree	6157 (76.1)	25.0	38.5	42.1
P		0.545	0.098	0.012*
**Mother watch tv**				
Disagree	4946 (61.1)	28.3	33.0	39.8
Agree	3146 (38.9)	21.7	45.5	40.6
P		0.003*	< 0.001*	0.758
**Mother surf internet**				
Disagree	7431 (91.8)	27.4	37.6	40.2
Agree	661 (8.2)	15.0	25.7	23.1
P		< 0.001*	0.152	0.210
**Health insurance coverage**				
Disagree	1523 (18.8)	17.9	28.2	31.6
Agree	6569 (81.2)	25.9	39.1	42.3
P		0.010*	0.001*	< 0.001*
**Mother’s marital status**				
Single	719 (8.9)	16.4	23.3	28.7
Currently in union/living with a man	6734 (83.2)	25.9	39.5	41.2
Formerly in union	639 (7.9)	19.1	30.8	34.3
P		0.030*	< 0.001*	0.033*
**Mother currently working**				
Disagree	2009 (24.8)	25.5	31.6	36.5
Agree	6083 (75.2)	24.1	39.1	40.8
P		0.510	0.010*	0.185
**Sex of household head**				
Male	6257 (77.3)	26.6	38.2	40.5
Female	1835 (22.7)	18.8	34.0	37.8
P		0.003*	0.167	0.428
**Household wealth index**				
Poorest	2100 (26.0)	24.5	34.9	38.7
Poorer	1666 (20.6)	27.0	38.4	44.6
Middle	1568 (19.4)	26.9	38.7	40.1
Richer	1443 (18.8)	27.8	39.9	40.2
Richest	1315 (16.3)	18.8	34.4	30.6
P		0.039*	0.550	0.093
**Geographical region**				
Kigali	948 (11.7)	13.7	27.5	0.0
South	1853 (22.9)	28.1	44.0	44.7
West	2069 (25.6)	42.9	46.8	49.3
North	1282 (15.8)	27.1	44.4	37.1
East	1940 (24.0)	14.7	17.2	27.5
P		< 0.001*	< 0.001*	< 0.001*
**Place of residence**				
Urban	1702 (21.0)	19.4	27.1	-
Rural	6390 (79.0)	31.3	37.7	40.0
P		< 0.001*	0.099	-
**Total estimate**	**8092 (100.0)**	**24.7**	**37.3**	**40.0**

* Significant at p < 0.05

**Fig. 1:**
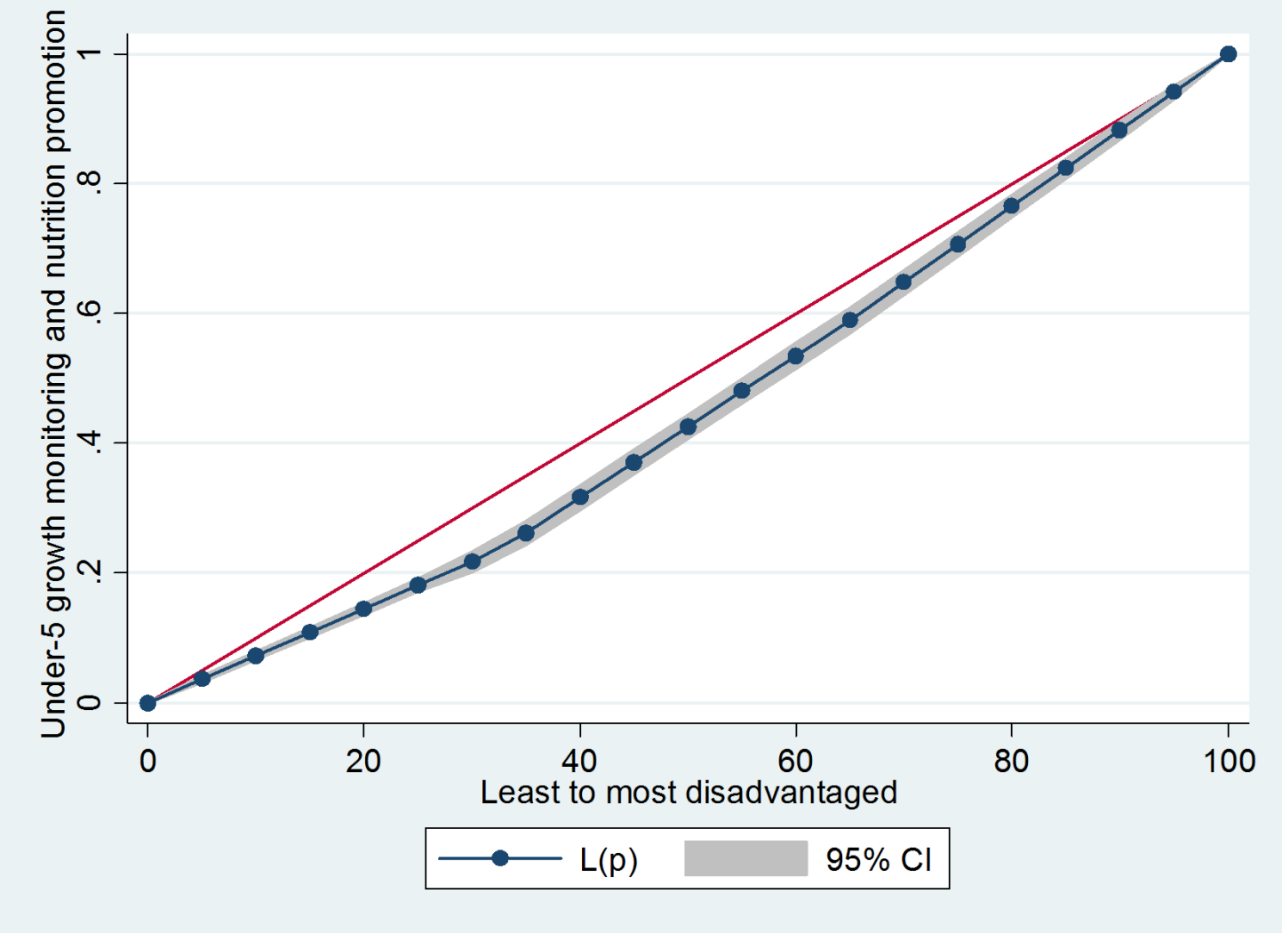
Lorenz curve for under-5 GMNP by socioeconomic disadvantaged level

The socioeconomic inequalities for under-5 GMNP in Rwanda are depicted in Fig. 1. How far the curves deviate from the line of equality indicates whether there are greater inequalities and to what extent. Figure 1 demonstrates that the most disadvantaged cohort had higher uptake of under-5 GMNP, as the line of equality sags below the diagonal line.

Table 2 showed results of socioeconomic disadvantaged inequalities for under-5 GMNP. Overall, there was pro-poor under-5 GMNP (Conc. Index = 0.0994; SE = 0.0111). Across the levels of child and mother’s characteristics, the results show higher coverage of under-5 GMNP in the most socioeconomic disadvantaged cohort. In addition, there was difference in the concentration indices across the levels of the following variables: family motility (p = 0.005), mothers who watch TV (p = 0.001), sex of household headship (p = 0.021) and geographical region (p = 0.005) respectively.

**Table 2:** Socioeconomic inequalities in under-5 GMNP

Variable	Concentration Index	Standard Error	P
**Child’s age (months)**			0.864
0–11	0.0993*	0.0213	
12–23	0.1113*	0.0179	
24–35	0.0775	0.0429	
36–47	0.0785*	0.0310	
48–59	0.0829*	0.0280	
**Preceeding birth interval**			0.797
< 24 months	0.0919*	0.0329	
24–48 months	0.0908*	0.0152	
> 48 months	0.0766*	0.0274	
First born	0.1104*	0.0248	
**Birthweight (kg)**			0.083
< 2.5	0.0253	0.0481	
≥ 2.5 (normal weight)	0.1057*	0.0117	
**Sex of child**			0.800
Male	0.1027*	0.0160	
Female	0.0970*	0.0155	
**Place of delivery**			0.489
Health facility	0.0988*	0.0114	
Home	0.1379*	0.0466	
**Mother’s age (years)**			0.117
15–24	0.0553	0.0297	
25–34	0.1173*	0.0158	
35+	0.0929*	0.0180	
**Family motility**			0.005*
< 5 years	0.1291*	0.0214	
5 + years (native)	0.0630*	0.0128	
**Mother’s education**			0.526
No formal education	0.0474	0.0241	
Primary	0.0731*	0.0136	
Secondary+	0.0937*	0.0249	
**Household size**			0.083
1–4	0.1030*	0.0195	
5–6	0.1212*	0.0169	
7+	0.0583*	0.0225	
**Mother read newspaper**			0.081
Disagree	0.0924*	0.0124	
Agree	0.1417*	0.0241	
**Mother listen to radio**			0.086
Disagree	0.0675*	0.0236	
Agree	0.1126*	0.0125	
**Mother watch tv**			0.001*
Disagree	0.0720*	0.0140	
Agree	0.1503*	0.0177	
**Mother surf internet**			0.915
Disagree	0.0743*	0.0112	
Agree	0.0679	0.0392	
**Health insurance coverage**			0.814
Disagree	0.1093*	0.0316	
Agree	0.1023*	0.0118	
**Mother’s marital status**			0.876
Single	0.1168*	0.0528	
Currently in union/living with a man	0.0956*	0.0116	
Formerly in union	0.1012*	0.0500	
**Mother currently working**			0.617
Disagree	0.0795*	0.0226	
Agree	0.0918*	0.0126	
**Sex of household head**			0.021*
Male	0.0855*	0.0121	
Female	0.1493*	0.0273	
**Household wealth index**			0.592
Poorest	0.0775*	0.0212	
Poorer	0.1000*	0.0224	
Middle	0.0810*	0.0248	
Richer	0.0844*	0.0252	
Richest	0.1313*	0.0323	
**Geographical region**			0.005*
Kigali	0.0563	0.0339	
South	0.0720*	0.0199	
West	0.0294	0.0164	
North	0.0406	0.0272	
East	0.1417*	0.0325	
**Place of residence**			0.725
Urban	0.0242	0.0168	
Rural	0.0405*	0.0114	
**Total**	**0.0994**	**0.0111**	< 0.001*

* Significant at p < 0.05; p = comparing concentration indices across the levels of a variable

Table 3 shows Erreygers concentration (Ec) indices decomposed in order to determine the contribution (Contri) of under-5 GMNP in Rwanda. Place of residence (Contri: 82.3650%, E_c_: 0.0633), mother internet use (Contri: 18.4502%, E_c_: -0.5608), household wealth (Contri: 15.7047%, E_c_: -0.0749), family motility (Contri: 10.8086%, E_c_: 0.0280), mother’s employment (Contri: 6.1858%, E_c_: 0.0686) and mother’s education (Contri: 5.2514%, E_c_: -0.1009) were positive contributors of under-5 GMNP. On the other hand, geographical region (Contri: -22.8640%, E_c_: 0.0610), mother watch tv (Contri: -7.3178%, E_c_: -0.1587) and mother listen to radio (Contri: -6.3425%, E_c_: -0.0522) were negative contributors to under-5 GMNP.

**Table 3:** Decomposition of under-5 GMNP uptake

Variable	Elasticity	Concentration Index	Absolute Contribution	% Contribution
Child’s age (months)	0.0468	0.0041	0.0008	0.5590
Preceeding birth interval	-0.0325	-0.0153	0.0020	1.4708
Birthweight (kg)	0.0433	-0.0015	-0.0003	-0.1927
Child’s sex	0.0262	-0.0040	-0.0004	-0.3124
Place of delivery	0.0027	0.3308	0.0035	2.6097
Mother’s age	0.0594	0.0041	0.0010	0.7257
Family motility	0.1309	0.0280	0.0147	10.8086
Mother’s education	-0.0176	-0.1009	0.0071	5.2514
Household size	-0.0543	0.0069	-0.0015	-1.1003
Mother read newspaper	0.0100	-0.1505	-0.0060	-4.4370
Mother listen to radio	0.0412	-0.0522	-0.0086	-6.3425
Mother watch tv	0.0156	-0.1587	-0.0099	-7.3178
Mother surf internet	-0.0112	-0.5608	0.0250	18.4502
Health insurance coverage	0.0886	-0.0166	-0.0059	-4.3481
Mother’s marital status	0.0344	0.0121	0.0017	1.2231
Mother’s employment	0.0305	0.0686	0.0084	6.1858
Sex of household head	-0.0745	-0.0169	0.0050	3.7098
Household wealth	-0.0711	-0.0749	0.0213	15.7047
Geographical region	-0.1270	0.0610	-0.0310	-22.8640
Place of residence	0.4411	0.0633	0.1117	82.3650

## Discussion

This is among the foremost studies in Rwanda to examine socioeconomic inequalities in under-5 GMNP. Similar to a previous research, the uptake of under-5 GMNP is low [25]. In addition, we found pro-poor GMNP among under-5 population. The key finding indicated that the most disadvantaged children had higher uptake of under-5 GMNP in Rwanda. This could be as a result of social and economic changes in resource-constrained settings which have experienced age-long developmental, epidemiological and demographic catastrophe. The socioeconomic distribution of health outcomes in several countries, has shifted in a way that has led to global health inequalities [34]. Notably, children aged 48–59 months that are most socioeconomically disadvantaged had greater uptake of under-5 GMNP, when compared with other age groups. This is in line with a recent study [25]. On the other hand, previous studies conducted in Ethiopia found children between 12 and 24 months to be more likely to utilize childhood GMNP services [12] [35]. Those studies however covered under 24 months old. It is well known that children from families with low socioeconomic status, have greater need for healthcare services. The poor health conditions of under-privileged and vulnerable children requires improved health management and maintenance [36]. The higher uptake of under-5 GMNP reported among the socioeconomically disadvantaged population reflects the higher probability of older children experiencing greater food insecurity and could be malnourished.

This paper is first to explore socioeconomic inequalities in the uptake of under-5 GMNP using concentration index and Lorenz curves. We found differences in concentration indices across the levels of certain variables, such as family motility, mothers who watch TV, sex of household headship and geographical region respectively. We found considerable inequalities related to years lived in an area of residence. The degree of inequality in under-5 GMNP was wider for children who live in an area for less than five years, when compared with the native. This corroborates with a recent study from Rwanda that found that children from families who are native residents have higher uptake of under-5 GMNP [25]. A possible explanation could be that native residents may likely be more aware of the availability of under-5 GMNP services, than the non-natives. It is also possible that indigenous residents have better geographic access to these services.

In addition, the differences in regional coverage could be attributed to diverse interventions related to under-5 GMNP which may have been executed in various regions and in varied capacity or scale. A recent study found that the uptake of under-5 GMNP was higher in the southern, western and northern regions, when compared with children from Kigali [25]. Another study conducted in Rwanda have reported similar findings [37]. This disparities in the uptake of under-5 GMNP across regions could be that children who reside in the geographical regions with lower uptake, may be unaware of the services available or unable to attend the sessions due to economic or transportation challenges. Since this survey was conducted during the Coronavirus pandemic, it is possible that the uptake may have been disrupted by the pandemic, especially that some regions may have served as the epicenter of COVID-19. Thus indicating that more of this intervention is needed in these regions by promoting the coverage and supporting caregivers to present their children as when scheduled.

We found that the uptake of under-5 GMNP was significantly higher among children from mothers who watch TV, when compared with those from mothers do not watch TV. It could be that mothers who watch TV are more aware and enlightened about under-5 GMNP, than mothers who do not watch TV. It is known that mother’s exposure to mass media play an important role in enhancing health services uptake. Mothers who are expose to mass media are better informed about programmes that promote the health children as well as know about healthcare initiatives [34] [38].

The sex of household headship influenced the uptake of under-5 GMNP. We found that children from male headed household had higher uptake of under-5 GMNP. Conversely, the degree of inequality in under-5 GMNP was wider among children from female headed household, when compared with those from male headed households. This is in contrast with a recent study conducted in Rwanda that did not find any association between under-5 GMNP and sex of household head [25]. Women’s empowerment is still required to improve health services uptake in a patriarchal society. Women could have lower levels of education, less access to employment and consequently become socioeconomically disadvantaged [39]. Policies and strategies need to be design and implemented to empower women and increase their socioeconomic development.

We conducted further analysis to decompose selected child and mother’s characteristics related to under-5 GMNP. Based on our findings, place of residence was the largest contributor to inequality, contributing about 82.4% of inequality to the uptake of under-5 GMNP. Other important contributors to inequality included mother’s internet use, household wealth, family motility, mother’s employment and education. Certainly, these variables have been identified as significant factors to consider in designing polices to increase under-5 GMNP in resource-constrained settings such as Rwanda.

The findings from our study would play a vital role in shaping nutrition policies for under-5 children. These could be used in evidence-based policy formulation and implementation. We identified socioeconomic inequalities in the uptake of under-5 GMNP. Hence, policies can be tailored to address socioeconomic inequalities directly by promoting universal health coverage to reach all under-5 children in Rwanda, irrespective of their socioeconomic status. In addition, the findings can be used to design and implement effective nutrition education programmes for caregivers, parents, communities and empower local leaders to promote nutrition within their communities. These programmes can help raise awareness about proper nutrition and child growth, as well as promote healthy feeding practices. Moreover, stakeholders in healthcare system can use the findings of our study to implement nutrition policies with built-in evaluation mechanisms to regularly assess their effectiveness. Our findings can also guide in taking a comprehensive approach to addressing the nutritional and health needs of under-5 children including the uptake of growth monitoring, as it is possible to make significant improvements in their nutritional status and overall well-being by enhancing the socioeconomic development of the country at large. Furthermore, our study has brought to limelight, using a population-based data, the coverage and inequalities in under-5 GMNP in Rwanda. It is our hope that the findings of this study will be useful to stakeholders in healthcare for designing and implementing viable programmes that will help the socioeconomically disadvantaged cohort recover from child undernutrition in near future and address the disparities in prevalence of nutritional status even with the advantaged children.

### Strength and limitations

The use of recent nationally representative household survey data is a major strength of this study, and the findings are generalizable to under-5 children in Rwanda. The main outcome variable was, however, measured using self-reported data, which may have recall bias. Consequently, the uptake of under-5 GMNP may have been over- or underestimated. DHS did not obtain information on household income and expenditure. Therefore, asset-based wealth index was used in this study. In addition, variables on caregivers’ attitudes toward children’s health were not available because the study conducted secondary data analysis. Furthermore, because availability of under-5 GMNP sessions can affect attendance, we were unable to conduct an exhaustive assessment of this factor due to the use of secondary data, which had the flaw of not containing information on the availability of sessions for growth monitoring and nutrition promotion. Moreover, we found inadequate coverage of growth monitoring and nutrition promotion among under-five in Rwanda. As we conducted a secondary data analysis, there was no information regarding the growth monitoring system, whether it has sufficient manpower, infrastructure, standard operating procedures or demand creation strategies, which could influence the level of uptake. We relied on self-reported uptake of growth monitoring and nutrition promotion.

## Conclusion

This study demonstrated a pro-poor inequality in under-5 GMNP. The study showed that the most socioeconomically disadvantaged children had higher prevalence of under-5 GMNP. The findings show that individual socioeconomic characteristics such as place of residence, wealth status, maternal education, are contributors to improving inequality. Therefore, intervention policies should be centred on these elements to reduce the disparity in the uptake of under-5 GMNP. A further effective policy strategy for reducing socioeconomic inequalities in the practice of growth monitoring and promoting optimal nutrition could be helpful in healthcare system’s collaboration with other social and development sectors.

## Data Availability

Data for this study were sourced from Demographic and Health surveys (DHS) and available here: http://dhsprogram.com/data/available-datasets.cfm.
